# Dr. Loren L. Eddy

**Published:** 1900-04

**Authors:** 


Dr. Loren L. Eddy, U. of B., '97, of Olean, N. Y., died at his home, March 15, 1900, aged 29 years. He was the son of Dr. J. L. Eddy, who is one of the most prominent physicians of the Southern Tier, and the deceased himself was growing into prominence with great rapidity. He was a member of the 43d Separate Company N.Y.N.G., and was the medical officer of the company. His funeral was largely attended on Sunday, March 18th, and the burial was with military honors. Dr. Eddy married Miss Rosalie Baylor Freeman, of Buffalo, who, with an infant daughter, survives him.

